# Internal Transcribed Spacer 1 (ITS1) based sequence typing reveals phylogenetically distinct *Ascaris* population

**DOI:** 10.1016/j.csbj.2015.08.006

**Published:** 2015-09-04

**Authors:** Koushik Das, Punam Chowdhury, Sandipan Ganguly

**Affiliations:** Division of Parasitology, National Institute of Cholera and Enteric Diseases, P-33, CIT Road, Scheme XM, Beliaghata, Kolkata 700010, India

**Keywords:** Ascariasis, Ascaris sp, Genetic diversity, Phylogeny, Internal transcribed spacer 1 (ITS1), Sequence typing

## Abstract

Taxonomic differentiation among morphologically identical *Ascaris* species is a debatable scientific issue in the context of Ascariasis epidemiology. To explain the disease epidemiology and also the taxonomic position of different *Ascaris* species, genome information of infecting strains from endemic areas throughout the world is certainly crucial. *Ascaris* population from human has been genetically characterized based on the widely used genetic marker, internal transcribed spacer1 (ITS1). Along with previously reported and prevalent genotype G1, 8 new sequence variants of ITS1 have been identified. Genotype G1 was significantly present among female patients aged between 10 to 15 years. Intragenic linkage disequilibrium (LD) analysis at target locus within our study population has identified an incomplete LD value with potential recombination events. A separate cluster of Indian isolates with high bootstrap value indicate their distinct phylogenetic position in comparison to the global *Ascaris* population. Genetic shuffling through recombination could be a possible reason for high population diversity and frequent emergence of new sequence variants, identified in present and other previous studies. This study explores the genetic organization of Indian *Ascaris* population for the first time which certainly includes some fundamental information on the molecular epidemiology of Ascariasis.

## 1 Introduction

Human Ascariasis caused by gastrointestinal nematode *Ascaris lumbricoides* (L) is one of the major Soil Transmitted Helminthiases (STHs).The disease has been included in World Health Organization (WHO) list of Neglected Tropical Diseases (NTD), infecting more than one billion people [1]. Transmission is normally through the ingestion of infective *Ascaris* sp. egg in sewage contaminated soil and vegetables. Majority of infections are asymptomatic, while some chronic infection develops symptoms like abdominal pain, nausea, lung inflammation, anemia, stunted growth, diminished physical fitness etc. [1]. This has a certain impact on socio-economic development of low-income countries [2]. Like human, pigs are also infected with closely related species of *A*. *lumbricoides* (L), *Ascaris suum* Goeze [3]. Taxonomic separation between *A*. *lumbricoides* and *A*. *suum* represents a debatable scientific issue in the context of Ascariasis epidemiology due to the absence of distinguishing morphological characteristics among them [4]. Proper identification and genetic characterization of infecting strains from endemic areas throughout the world are certainly important to explain the disease epidemiology and also the taxonomic status of two *Ascaris* species. Several molecular epidemiological investigation based on polymorphic markers like-internal transcribed spacer 1 (ITS1), mitochondrial cytochrome c oxidase subunit 1 (cox1), NADH dehydrogenase subunit 1 (nad1) and microsatellite markers have been proposed to explain the origin of the two ascarid taxa in their respective hosts and their taxonomic status [5–8]. However, non-repetitive genomic regions have been preferred over repetitive regions as genotyping marker for their high genetic stability and evolutionary significance. Single nucleotide substitution occurred just once in the phylogenetic history of a species, unlikely to mutate again to either a novel or ancestral genotype [9]. Using the non-repetitive marker ITS1, 5 *Ascaris* genotypes (G1-G5) in human and 3 *Ascaris* genotypes (G1-G3) in pig have been identified. G1 frequently infects human, while G3 is predominant in pigs. The other three has been detected in lower frequencies in their respective hosts [5]. Recently, a study from Brazil based on ITS1 marker reported a new *Ascaris* genotype G6 in human [10]. However, no such information regarding genetic pattern and diversity of *Ascaris* population from India are available still date. Hence, the present study was designed to generate an idea about genetic patterns and diversity of Indian *Ascaris* population and also to determine their phylogenetic relation with the global *Ascaris* population. The result revealed a considerable amount of polymorphism within our *Ascaris* population. Along with the previously reported and widely distributed genotype G1 [5,10], 8 new sequence variants of ITS1 (IND1-IND8) have been identified. Since, *Ascaris sp*. multiply through sexual reproduction and genetic recombination during meiosis is a natural phenomenon [11], Efforts were also made to determine whether this population diversity are associated with genetic shuffling. Intragenic linkage disequilibrium (LD) among our study population was evaluated at ITS1 locus to identify potential recombination events within them. Moreover, any significant association of *Ascaris* genotypes with patient's age and sex was also evaluated.

## 2 Material and methods

### 2.1 Sample collection and detection of Ascaris sp.:

A total of 35 *Ascaris* isolates from human were included in our study. Fecal samples were collected from people of “low socio-economic community of Kolkata” through an on-going field project, studying the parasite burden of those communities. Poor hygiene, sanitation and malnutrition were common in those communities [12]. The ethical clearance for this study has been provided by NICED IEC (i.e. National Institute of Cholera and Enteric Diseases Institutional Ethical Committee). Informed consents have been obtained from the patients (in case of children consents have been obtained from their parents). The parasite's eggs within the fecal were primarily detected by microscopy [13]. DNA was isolated directly from microscopy positive fecal samples using STOOL DNA Minikit (QIAGEN, USA) as per manufacturer's protocol.

### 2.2 Polymerase chain reaction (PCR) amplification and DNA sequencing

Partial amplification of target gene (ITS1) was performed using gene specific primer pairs (Table 1). In all cases the PCR reaction was performed in 50 μl reaction volume containing approximately 0.4 μg and 0.1 μg of template DNA for primary and nested PCR respectively, 10 pM of each primer, 2.5 mM MgCl_2_, 1 μg of Bovine Serum Albumin (SIGMA, USA), 200 μM dNTP and 2.5 U of *Taq* DNA polymerase (Bioline, USA) with the reaction parameters as initial denaturation for 5 min or 4 min (Primary and Nested respectively) at 94 °C. This was followed by 35 cycles of denaturation at 94 °C for 30 s, annealing at 65 °C or 60 °C (Primary and Nested respectively) for 30 s, extension at 72 °C for 30 s. This was again followed by the final extension for 10 min at 72 °C. The amplified PCR products were then separated by electrophoresis on 1.5% agarose gels (SIGMA, USA) according to their size. PCR products of expected sizes were extracted from gels and purified (ROCHE, Germany). The Purified PCR products were then sequenced directly with specific primers (marked with ^a^ in Table 1) using the ‘BigDye Terminator V3.1 cycle sequencing kit’ (APPLIED BIOSYSTEMS, USA) as per the manufacturer's protocol. The labeled DNA fragments were further purified by sodium acetate and ethanol precipitation. The sequencing was carried out in an ABI 310 PRISM Automated Genetic Analyzer. Accuracy of DNA sequencing data has been confirmed by sequencing in both directions and also by repetition of DNA sequencing with a new PCR product for all study isolates.

### 2.3 Analysis of sequence polymorphisms

ITS1 sequences of our study isolates were aligned with all previously published sequences of corresponding loci (downloaded from NCBI GenBank database, accession numbers have been provided in Table 2) using ClustalW multiple alignment program of MEGA version 4 software [14]. Nucleotide position of each single nucleotide polymorphism (SNP) within the target loci was identified from the aligned sequences. The nucleotide positions of SNPs within the target loci were relative to the reference sequence of G1 genotype (GenBank accession number AJ554036) (Table 2). Variable sequences of our target loci (in respect to the reference sequence) were submitted to NCBI GenBank database with accession numbers JN176638 – JN176674.

Intragenic LD and number of recombination events at ITS1 locus among our study population were also assessed by using DnaSP version 5.10.01 (www.ub.es/dnasp/) software.

### 2.4 Statistical analysis

Associations of *Ascaris* genotypes with patient's age and sex have been evaluated by Epi-Info version 3.5.4 software [15].

### 2.5 Phylogenetic analysis

Phylogenetic trees were constructed from the previously aligned ITS1 sequences by MEGA version 4 software [14]. Two individual methods [i.e. Neighbor-Joining (NJ) and Maximum parsimony (MP)] were used to confirm the topology of the tree. In both cases, widely distributed and most prevalent *Ascaris* genotype G1 (Genbank ID AJ554036) was considered as an out-group. The bootstrap values were also analyzed to estimate confidence intervals. Genetic distance analysis among our study isolates was also performed using MEGA version 4 software [14].

## 3 Results

Among our 35 study isolates, majority (27) corresponded to previously reported and widely distributed genotype G1 [5,10]. Along with genotype G1, 8 new sequence variants of ITS1 have also been identified. These new sequence variants have assigned alphanumerical codes beginning with letter ‘IND’ to indicate their Indian origin (i.e. IND1-IND8) (Table 2).

**Fig. 1:**
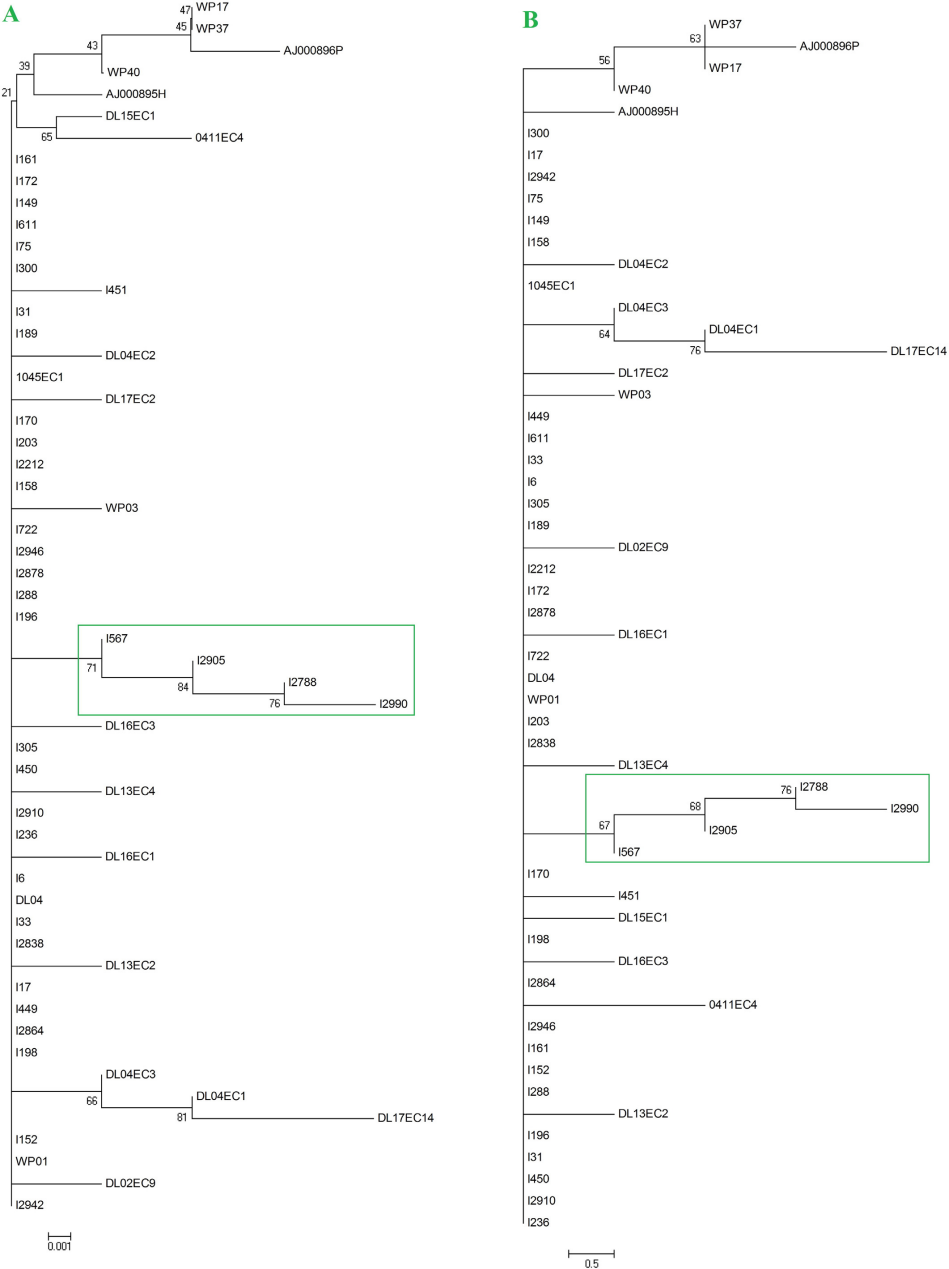
Phylogenetic comparison of our study isolates with the global *Ascaris* population: Internal transcribed spacer 1 (ITS1) sequences of our study isolates were aligned with ITS1 sequences from the global *Ascaris* population using ClustalW multiple alignment program of MEGA version 4 software. Two separate phylogenetic trees were generated from this alignment using two individual methods- [A] Neighbor-Joining (NJ) and [B] Maximum parsimony (MP). In both cases, widely distributed and most prevalent *Ascaris* genotype G1 (Genbank ID AJ554036) was considered as out-group. The bootstrap values were also analyzed to estimate confidence intervals. Phylogenetic comparison using these two individual methods (i.e. NJ and MP) generates trees with similar topology. In both cases, few of our Indian isolates (with newly identified ITS1 sequences) formed distinct cluster with high bootstrap value (marked with green color in both trees), which may indicates their distinct phylogenetic position in respect to global *Ascaris* population.

Sequence comparison of our study isolates with the global *Ascaris* population has revealed 17 new SNPs, which were present in 8 newly identified ITS1 sequences from our Indian *Ascaris* population (i.e. IND1-IND8) (Table 2). Phylogenetic comparison of our study isolates with the global *Ascaris* population using two individual methods (i.e. NJ and MP) generates trees with similar topology. In both cases, few of our Indian isolates (with newly identified ITS1 sequences) formed distinct cluster with high bootstrap value (marked with green color in both trees), which may indicates their distinct phylogenetic position in respect to global *Ascaris* population (Fig. 1). We have also performed genetic distance analysis among our study isolates. The result has been provided in data 1. Intragenic LD between pairs of polymorphic sites at ITS1 locus of our study population was also evaluated to identify potential recombination events within them. Among 171 pairwise comparisons, 92 were significant by Chi-square test and 89 were significant after Bonferroni correction (Table 3). An incomplete LD value (|D′| Y = 0.9818 + 0.1974X, where Y is the LD value and X is the nucleotide distance in kilobases) was also detected (Table 3). Moreover, intragenic recombination analysis at ITS1 locus of our study isolates has identified 2 potential recombination events within our study population (Table 3). Moreover, any significant association of *Ascaris* genotypes with patient's age and sex was also studied, which revealed that G1 genotype was significantly present among female patients (co-efficient value = 0.815, p value = 0.000002) aged between 10 to 15 years (co-efficient value = 0.690, p value = 0.000105). The age and sex information of the patients, included in our study has been provided in Table 4.

## 4 Discussion

*A*. *lumbricoides* and *A*. *suum* are two of the world's most common soil transmitted nematode and together cause serious health and socio-economic problems. Ascariasis has been considered as Neglected Tropical Diseases (NTD) by WHO, since it is highly prevalent in poor urban and rural areas and has a certain impact on patient's health, physical fitness and productivity [1,2]. Morphological similarity of these two nematodes entails ongoing uncertainty concerning their taxonomic status and argues for the need to explore deeper into their molecular epidemiology [4]. A recent surveillance study among school children from south India revealed a highest prevalence of *Ascaris* species among all STHs infections. Co-infection with other STHs has also been reported [16]. Even though few surveillance studies on *Ascaris* infection have been conducted in India, diagnosis of this parasite was solely based on microscopy. Differentiation between *Ascaris* species certainly cannot be confirmed by microscopy but require detailed molecular epidemiological study based on genetic markers. In the present study, *Ascaris* population from human has been genetically characterized based on widely used genetic marker ITS1.

Sequence analysis of our study isolates has identified G1 as a dominant genotype. As much as 27 among 35 study isolates were corresponding to this widely distributed genotype. This result corroborates with previous report from China, where genotype G1 was dominant among human and G3 among pig [5]. Our study has also identified 8 new sequence variants of ITS1 (IND1-IND8) within our Indian *Ascaris* population. Similar finding was previously reported by Leles *et*.*al* from Brazil. They have also identified 13 new *Ascaris* haplotypes (H1–H13) from human [17]. Sequence comparison of our study isolates with previously reported *Ascaris* sequences has identified 17 new SNPs within our study isolates. Moreover, all of these SNPs were present within 8 newly identified sequence variants of ITS1 (IND1–IND8), which indicates their distinct genetic organization. This finding was further well supported by the observation of phylogenetic analysis. All the previously reported *Ascaris* sequences were retrieved from NCBI database and *Ascaris* sequences from our study isolates were phylogenetically compared with them. Phylogenetic analysis revealed an interesting scenario. Few of our Indian isolates (with new variations of ITS1 sequences) formed a separate cluster with high bootstrap value, indicating their distinct phylogenetic position in respect to the global *Ascaris* population. Moreover, Intragenic LD analysis between pairs of polymorphic sites at ITS1 locus has identified an incomplete LD value with two potential recombination events within our study population. This finding was quite compatible with a previous report by Li *et*.*al*. They have identified a similar type of observation (presence of intragenic LD value and recombination events) in *gp60* locus of another enteric parasite, *Cryptosporidium homonis* [18]. Since, *Ascaris sp*. multiply through sexual reproduction [11], genetic recombination during meiosis could be a natural phenomenon. Furthermore, a recent study has identified the molecular evidence of polyandry in *A*.*suum*. Single female of *A*. *suum* can mate with multiple males, which can also increase the chance of genetic variations [19]. Such high possibilities of genetic shuffling could be associated with increasing population diversity in a restricted geographic region [5,7,10,17] and frequent emergence of new sequence variants, identified in present as well as in previous studies [17]. Attempts were also made to determine whether any statistically significant association exists between the identified *Ascaris* genotypes and patient's age and sex. Genotype G1 was found to be significantly present among female patients (co-efficient value = 0.815, p value = 0.000002) aged between 10 to 15 years (co-efficient value = 0.690, p value = 0.000105). This finding was quite congruous with a previous report by Anuar *et*.*al* [20]. They have reported that Ascariasis was significantly related to patients aged < 15 years and earning low household income.

Since, Ascariasis is one of the major Soil Transmitted Helminthiases (STHs) and has been declared as Neglected Tropical Diseases (NTD) by WHO, genome information of its infecting strains from different parts of the world is certainly crucial to investigate the disease epidemiology. This study explores the genetic organization of Indian *Ascaris* population for the first time; it will certainly include some fundamental information on the molecular epidemiology of Ascariasis.

## Figures and Tables

**Table 1:** List of gene specific primers, used in the study.

Gene name	PCR round	Primer name	Primer sequence (5′ to 3′)
Internal transcribed spacer 1 (ITS1)	Primary	ITS F 1	CGAGCAGAAAAAAAAAAGTCTCC
ITS R1	GGAATGAACCCGATGGCGCAAT
Secondary	ITS F 2^a^	CGAGCAGAAAAAAAAAAAAGTCTCC
ITS R 2^a^	GCTGCGTTCTTCATCGAT

a Gene specific primer pairs used for sequencing of amplified PCR products.

**Table 2:** List of polymorphic sites identified within internal transcribed spacer 1 (ITS1) region among various *Ascaris* isolates from India and Worldwide.

Sample ID	GenBank accession number	Genotypes/haplotypes	Country	Host	Nucleotide variation at alignment position^a^	References
WP01	AJ554036	G1^b^	Ba^c^/Br^d^/Ja^e^/Ch^f^	Hu^g^/P^h^	120T, 122T, 123T, 124T, 125T, 127T, 128--i, 129 --i, 130G, 131C, 132G,133G, 134A, 135C, 139T, 142A, 148 --i, 150T, 151T, 155A, 156T, 157--i, 162T, 167A, 168A, 169G, 172T, 173G, 176T, 181T, 183T, 203G, 205C, 206G, 207C, 218T, 229T, 231C, 233T, 238A, 248T	[5,10,21]
WP40	AJ554037	G2^b^	Ch^f^	Hu^g^/P^h^	128--i/T, 133G/S^j^	[5]
WP17	AJ554038	G3^b^	Ja^e^/Ch^f^	Hu^g^/P^h^	128--i/T, 133G/C, 248T/A	[5,21]
WP03	AJ554039	G4^b^	Ch^f^	Hu^g^	173G/R^k^	[5]
WP37	AJ554040	G5^b^	Ch^f^	Hu^g^	133G/S^j^, 248T/W^l^	[5]
DL04	EF153621	G6^b^	Br^d^	Hu^g^	120T/--i	[10,17]
AJ000895H	AJ000895	Al^m^	Au^n^	Hu^g^	205C/S^j^	[7]
AJ000896P	AJ000896	As^o^	UK^p^/De^q^	P^h^	128--i/T, 129--i/T, 133G/C, 205C/S^j^, 248T/A	[7]
DL02Ec9	GQ339794	H1^b^	Br^d^	Hu^g^	203G/A	[17]
DL04Ec1	EU635686	H2^b^	Br^d^	Hu^g^	124T/--i, 127T/--i, 156T/C, 231C/T	[17]
DL04Ec2	EU635687	H3^b^	Br^d^	Hu^g^	127T/--i, 183T/A	[17]
DL04Ec3	EU635688	H4^b^	Br^d^	Hu^g^	156T/C, 127T/--i	[17]
DL13Ec2	GQ339795	H5^b^	Br^d^	Hu^g^	167A/G, 120T/--i	[17]
DL13Ec4	GQ339796	H6^b^	Br^d^	Hu^g^	150T/C	[17]
DL15Ec1	GQ339797	H7^b^	Br^d^	Hu^g^	120T/--i, 229T/C	[17]
DL16Ec1	GQ339798	H8^b^	Br^d^	Hu^g^	120T/C, 233T/C	[17]
DL16Ec3	GQ339799	H9^b^	Br^d^	Hu^g^	124T/--i, 218T/C	[17]
DL17Ec2	EU635694	H10^b^	Br^d^	Hu^g^	127T/--i, 238A/G	[17]
DL17Ec14	EU635695	H11^b^	Br^d^	Hu^g^	127T/--i, 130G/T, 155A/G, 156T/C, 231C/T	[17]
041-1Ec4	GQ339800	H12^b^	Br^d^	Hu^g^	120T/--i, 229T/A, 248T/C	[17]
104-5Ec1	GQ339801	H13^b^	Br^d^	Hu^g^	124T/C	[17]
I158	JN176638	G1^b^	IND^r^	Hu^g^	I^s^	[*]^t^
I305	JN176639	G1^b^	IND^r^	Hu^g^	I^s^	[*]^t^
I300	JN176640	G1^b^	IND^r^	Hu^g^	I^s^	[*]^t^
I152	JN176641	G1^b^	IND^r^	Hu^g^	I^s^	[*]^t^
I6	JN176642	G1^b^	IND^r^	Hu^g^	I^s^	[*]^t^
I172	JN176643	G1^b^	IND^r^	Hu^g^	I^s^	[*]^t^
I149	JN176644	G1^b^	IND^r^	Hu^g^	I^s^	[*]^t^
I31	JN176645	G1^b^	IND^r^	Hu^g^	I^s^	[*]^t^
I203	JN176646	G1^b^	IND^r^	Hu^g^	I^s^	[*]^t^
I170	JN176647	G1^b^	IND^r^	Hu^g^	I^s^	[*]^t^
I449	JN176648	G1^b^	IND^r^	Hu^g^	I^s^	[*]^t^
I450	JN176649	G1^b^	IND^r^	Hu^g^	I^s^	[*]^t^
I2878	JN176655	G1^b^	IND^r^	Hu^g^	I^s^	[*]^t^
I198	JN176658	G1^b^	IND^r^	Hu^g^	I^s^	[*]^t^
I189	JN176659	G1^b^	IND^r^	Hu^g^	I^s^	[*]^t^
I33	JN176660	G1^b^	IND^r^	Hu^g^	I^s^	[*]^t^
I2212	JN176663	G1^b^	IND^r^	Hu^g^	I^s^	[*]^t^
I2946	JN176664	G1^b^	IND^r^	Hu^g^	I^s^	[*]^t^
I236	JN176665	G1^b^	IND^r^	Hu^g^	I^s^	[*]^t^
I2910	JN176666	G1^b^	IND^r^	Hu^g^	I^s^	[*]^t^
I17	JN176667	G1^b^	IND^r^	Hu^g^	I^s^	[*]^t^
I2864	JN176668	G1^b^	IND^r^	Hu^g^	I^s^	[*]^t^
I2838	JN176669	G1^b^	IND^r^	Hu^g^	I^s^	[*]^t^
I2942	JN176670	G1^b^	IND^r^	Hu^g^	I^s^	[*]^t^
I611	JN176672	G1^b^	IND^r^	Hu^g^	I^s^	[*]^t^
I75	JN176673	G1^b^	IND^r^	Hu^g^	I^s^	[*]^t^
I161	JN176674	G1^b^	IND^r^	Hu^g^	I^s^	[*]^t^
I451	JN176651	IND1^u^	IND^r^	Hu^g^	142A/C, 148--i/G, 157--i/T	[*]^t^
I2990	JN176653	IND2^u^	IND^r^	Hu^g^	130G/A, 131C/G, 173G/C	[*]^t^
I2788	JN176654	IND3^u^	IND^r^	Hu^g^	122T/A	[*]^t^
I288	JN176656	IND4^u^	IND^r^	Hu^g^	162T/--i	[*]^t^
I196	JN176657	IND5^u^	IND^r^	Hu^g^	120T/A	[*]^t^
I2905	JN176661	IND6^u^	IND^r^	Hu^g^	122T/A, 123T/A, 124T/A, 125T/A, 131C/G, 132G/A, 134A/G, 135C/A, 151T/G, 168A/T, 169G/T, 172T/A, 181T/G	[*]^t^
I567	JN176662	IND7^u^	IND^r^	Hu^g^	134A/G	[*]^t^
I722	JN176671	IND8^u^	IND^r^	Hu^g^	139T/--i	[*]^t^

a Numbers correspond to nucleotide position on reference sequence AJ554036[5].

b Previously reported *Ascaris* genotypes (G1– G6)/haplotypes (H1– H13).

c Ba: Bangladesh.

d Br: Brazil.

e Ja: Japan.

f Ch: China.

g Hu: human.

h P: pig.

i --: nucleotide deletion.

j S: nucleotide G or C.

k R: nucleotide A or G.

l W: nucleotide A or T.

m Al: *A*. *lumbricoides* (without nomenclature).

n Au: Australia.

o As: *A*. *suum* (without nomenclature).

p UK: United Kingdom.

q De: Denmark.

r IND: India.

s I: similar to reference sequence G1.

t [*]: identified in this present study.

u New sequence variants of ITS1, identified in our study.

**Table 3:** Intragenic linkage disequilibrium (LD) and recombination analysis at internal transcribed spacer 1 (ITS1) region of our study isolates.

Population	Number of samples analyzed	Number of polymorphic sites analyzed	Number of pairwise comparisons	Number of significant pairwise comparisons a	Intragenic LD(|D′|) b	Rm c
Indian	35	21	171	92(89)	Y = 0.9818 + 0.1974X	2

a Number of significant pairwise comparisons by Chi square test (after Bonferroni correction).

b Intragenic linkage disequilibrium (LD), where Y is the LD value and X is the nucleotide distance in kilobases.

c Minimum number of intragenic recombination events.

**Table 4:** Age and sex information of patients, included in the study.

Sample ID	Patient information		GenBank accession number	Genotypes/haplotypes
Age (Y:M)	Sex (M/F)
I158	10.2	M	JN176638	G1^a^
I305	12.6	F	JN176639	G1^a^
I300	10.7	F	JN176640	G1^a^
I152	14.11	F	JN176641	G1^a^
I6	10.7	F	JN176642	G1^a^
I172	12.5	F	JN176643	G1^a^
I149	12.1	F	JN176644	G1^a^
I31	15.0	F	JN176645	G1^a^
I203	12.6	F	JN176646	G1^a^
I170	11.8	M	JN176647	G1^a^
I449	11.7	F	JN176648	G1^a^
I450	13.9	F	JN176649	G1^a^
I2878	15.10	F	JN176655	G1^a^
I198	27.1	F	JN176658	G1^a^
I189	14.5	M	JN176659	G1^a^
I33	12.6	F	JN176660	G1^a^
I2212	26.9	M	JN176663	G1^a^
I2946	9.7	M	JN176664	G1^a^
I236	14.4	F	JN176665	G1^a^
I2910	10.9	F	JN176666	G1^a^
I17	10.1	F	JN176667	G1^a^
I2864	11.3	F	JN176668	G1^a^
I2838	13.2	F	JN176669	G1^a^
I2942	12.8	F	JN176670	G1^a^
I611	10.8	F	JN176672	G1^a^
I75	15.4	F	JN176673	G1^a^
I161	14.3	F	JN176674	G1^a^
I451	8.0	F	JN176651	IND1^b^
I2990	6.5	M	JN176653	IND2^b^
I2788	9.7	M	JN176654	IND3^b^
I288	12.7	F	JN176656	IND4^b^
I196	9.2	M	JN176657	IND5^b^
I2905	7.6	M	JN176661	IND6^b^
I567	37.5	F	JN176662	IND7^b^
I722	23.5	F	JN176671	IND8^b^

a Previously reported *Ascaris* genoype G1, identified in our study.

b New sequence variants of target locus (i.e. ITS1), identified in our study.

## Appendix A Supplementary data

Supplementary material Genetic distance analysis among our study isolates using MEGA version 4 software.
